# The koala (*Phascolarctos cinereus*) prostate: a proteomic perspective on gland segmentation

**DOI:** 10.1093/biolre/ioaf209

**Published:** 2025-09-12

**Authors:** Yolande Campbell, Stephen D Johnston, Chiara Palmieri, Taylor Pini

**Affiliations:** School of Environment, The University of Queensland, Gatton, Queensland, 4343, Australia; School of Veterinary Science, The University of Queensland, Gatton, Queensland, 4343, Australia; School of Environment, The University of Queensland, Gatton, Queensland, 4343, Australia; School of Veterinary Science, The University of Queensland, Gatton, Queensland, 4343, Australia; School of Veterinary Science, The University of Queensland, Gatton, Queensland, 4343, Australia; School of Veterinary Science, The University of Queensland, Gatton, Queensland, 4343, Australia

**Keywords:** prostate, koala, proteomics

## Abstract

Australia’s most iconic animal, the koala (*Phascolarctos cinereus*), faces significant population decline and while conservation has focused on assisted breeding technology and reproductive pathologies, koala reproductive biology remains poorly understood. In particular, the koala (*P. cinereus*) prostate has a vital role in the production of seminal plasma and facilitating successful reproduction. Furthermore, prostatitis regularly occurs as a consequence of chlamydiosis, a substantial factor in the ongoing decline of koala populations. Despite this, little is known about the koala prostate’s molecular physiology. This study presents the first proteomic profile of the koala prostate, offering insights into its histological segmentation and broader functional significance. Prostatic tissue was collected from six mature male koalas, with samples taken from the anterior and posterior segments. Proteins were digested using filter-aided sample preparation and analysed via liquid chromatography–tandem mass spectrometry with Zeno-SWATH acquisition. Peptide spectra were processed using DIA-NN and evaluated in RStudio to identify differentially expressed proteins and compare the koala prostate proteome with those of other species. Functional annotation and pathway analysis revealed that whole prostate activity was primarily centered on protein translation and muscle function. Segment-specific proteomic profiles demonstrated slight proteomic differentiation, with secretory proteins contributing to segment-specific functions. Cross-species comparisons showed strong homology between the koala and human prostate proteomes. This proteome provides a foundation for future investigations into prostate-related pathologies in koalas. Furthermore, understanding the koala prostate at a molecular level helps advance wildlife conservation through a better comprehension of its role in male fertility and offers broader evolutionary insight into marsupial reproduction.

## Graphical Abstract

**Graphical Abstract:**
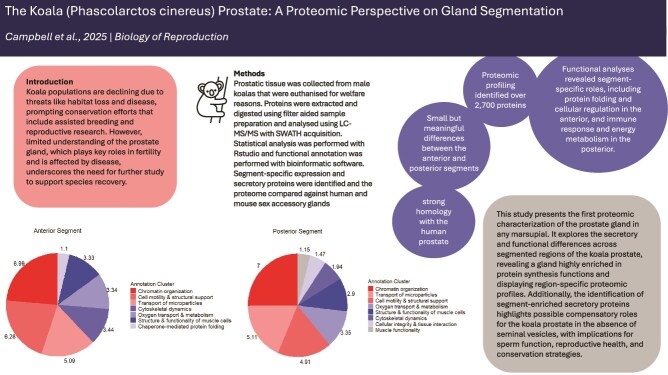

## Introduction

Australia is home to the largest and most diverse collection of marsupials and is the sole native habitat of the most iconic of these animals, the koala (*Phascolarctos cinereus*). Unfortunately, the koala is currently classified as vulnerable, with its population continuing to decline [1]. This decline is driven by habitat fragmentation and deforestation [2], vehicle strikes, animal attacks and the diseases chlamydiosis and koala retrovirus infection [3, 4]. Key conservation responses in this species have focused on the development and application of assisted breeding technology [5] and understanding the presentation, epidemiology, treatment and transmission of reproductive pathologies [6, 4]. The success of both conservation aims depends on a strong underpinning knowledge of koala reproductive biology.

Despite considerable research effort, many aspects of the koala’s reproductive biology still remain poorly understood; this is particularly true of their accessory sex glands. These glands have an important role in the production of mammalian seminal plasma required for successful reproduction and included the seminal vesicles, bulbourethral glands, ampullae and the prostate gland [7]. In mammals that have seminal vesicles, these glands produce the majority of seminal plasma [8]. Marsupials, however, lack seminal vesicles and possess only bulbourethral glands and a prostate [9]. It has been theorized that the marsupial prostate is responsible for the greatest contribution to seminal plasma [10]. The marsupial prostate is also considerably larger relative to body size than that of eutherian mammals [10]. Additionally prostate glands of marsupials demonstrate a significant degree of structural segmentation, with distinct regions of the glandular epithelium characterized by variations in cellular architecture and secretory morphology [11]. It is suggested that this segmentation arose as an adaptation, enabling the marsupial prostate to produce different constituents of seminal plasma [11]. This idea emphasizes the significant role of the marsupial prostate gland in their reproductive biology and suggests that this gland is highly derived in terms of evolutionary biology of this taxon.

The prostate gland of the koala is the most extensively researched sex accessory gland among marsupials, as seen in Table 1 of Campbell et al [12]. Johnston and Holt [9] have suggested that the prostate is responsible for the majority of seminal plasma whereas the primary role of the bulbourethral glands is to produce a copulatory plug. It has been suggested that the semen plasma, and therefore prostatic fluid, has a key role in contributing to induced ovulation in the female [13]. Furthermore, it also possible that koala prostatic fluid serves an important role in the successful preservation of spermatozoa. Research has demonstrated that an increased dilution of the koala ejaculate, including prostatic fluid, results in a corresponding increase in the percentage of nonlinear motility of koala spermatozoa [14]. Additionally, the unusually high survival of chilled koala spermatozoa for up to 42 days has been attributed to the integrity of prostatic bodies, although this relationship has yet to be confirmed [14]. Consequently, koala prostatic fluid may play a key role in the development of assisted breeding technology such as cryopreservation techniques and the development of a koala sperm bank [15].

**Table 1:** Functions of the secretory proteins that are enriched in the anterior and PSs of the koala prostate.

Koala prostate AS			Koala prostate PS		
Protein	Gene ID	Function	Protein	Gene ID	Function
A0A6P5LLT2	NUCB1	Calcium binding in the Golgi [42]	A0A6P5ICW3	GLA	Catalyzes the breakdown of glycoproteins and glycolipids [47]
A0A6P5KE16	LOC110208920	Function or homolog not yet known	A0A6P5IDH6	LOC110192427	Function or homolog not yet known
A0A6P5K7B0	LOC110207506	Function or homolog not yet known	A0A6P5IN09	ASPN	In the human prostate, regulates receptors in inflammatory responses, biomarker in prostate cancer [48]
A0A6P5LV82	ECM1	Involved in endochondral bone formation. Linked to the proliferation of endothelial cells and implicated in regulating the basement membrane [43]	A0A6P5IPM7	QPCT	Catalyst in formation of in numerous bioactive peptides and proteins [49]
A0A6P5JCR7	CPE	Sorting receptor in the regulated secretory pathway and processes prohormones in neuroendocrine cells [44]	A0A6P5IW93	CTSD	A protease that initiates breakdown of intracellular proteins, degradation of hormones and growth factors and regulates cell death [50]
A0A6P5LBW0	AGR2	A endoplasmic protein that is crucial for production of mucins and may have a role in cell proliferation [45]	A0A6P5J5M8	PAM	Enzyme that is critical to the biosynthesis of peptides, commonly present in neuroendocrine cells in the prostate [51]
A0A6P5IDD9	FAM174B	Membrane protein for Golgi structural integrity that may also have a role in vesicular transport [46]	A0A6P5J616	TNXB	An extracellular matrix glycoprotein with functions in tissue integrity and regulating collagen fibrils [52]
			A0A6P5K0C1	NAGA	An enzyme that cleaves glycopeptides and glycolipids [53]
			A0A6P5KXN8	LOC110214227	Function or homolog not yet known
			A0A6P5LAU5	F3	Transmembrane glycoprotein that initiates coagulation cascade. Also involved in cellular proliferation and development of blood vessels [54]
			A0A6P5LBL7	HEXB	Involved in the breakdown of glycoconjugates [55]
			A0A6P5LI86	ENDOD1	A nuclease that hydrolyses both DNA and RNA. Possibly functions as a tumor suppressor in prostate cancer [56]
			A0A6P5M1T5	EDIL3	An extracellular matrix protein involved in embryonic development, angiogenesis, and anti-inflammatory responses. Suggested role in tumorigenesis [57]

Beyond reproduction, the koala prostate has been highly implicated in pathology. Prostatitis, occurring as a consequence of chlamydiosis, is the leading cause of disease in male koalas [16]. Given the high prevalence of chlamydial bacteria within the prostate, this gland has been theorized as a potential reservoir for the infection [16]. The important and diverse roles of this gland reinforce the underlying need for a better understanding of how the koala’s prostate functions to drive applied outcomes for both captive breeding and conservation medicine.

**Figure 1:**
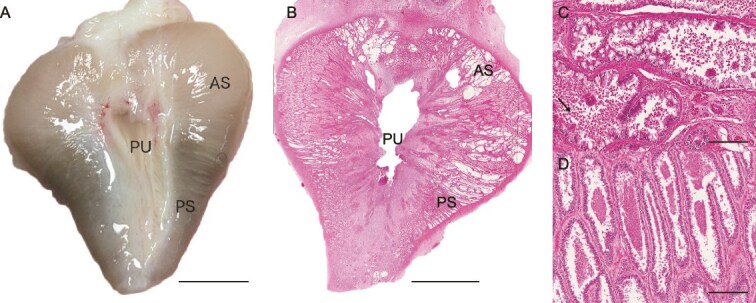
Gross and histological examination of the koala prostate (images sourced from authors). (A) Gross anatomical view showing the AS, PS, and prostatic urethra (PU) (bar = 6 mm). (B) Hematoxylin and eosin (H&E) stained section illustrating the overall gland with the AS, CS, PS and PU labeled (bar = 6 mm). (C) H&E staining of the AS of the koala prostate depicting stroma, epithelial tissue and globular secretions indicated by the arrow (bar = 200 μm). (D) H&E-stained section of the CS depicting stromal and epithelial tissue with intraluminal secretory material (bar = 700 μm). (E) H&E stained section of the PS demonstrating stromal and epithelia tissue with intraluminal secretions (bar = 200 μm).

Like all marsupials [11], koalas exhibit segmentation in the glandular epithelium of the prostate gland [17]. The anterior, central and posterior segments of the koala prostate gland differ significantly in term of their microanatomy and cellular architecture as depicted by histological and immunohistochemical studies [12] (Figure 1). The secretions produced by each segment are also morphologically and histochemically distinct [12] (Figure 1). However, it remains unclear whether there is any functional purpose to the segmentation in the koala’s prostate gland. Although the proteome of the koala ejaculate has been described by Skerrett-Byrne et al. [15] and histochemistry of the koala prostate points to functional segmentation [12], there has not yet been an in-depth molecular investigation of koala prostatic tissue.

The aim of this study was to further characterize segmentation of the koala prostate through proteomic analysis in attempt to explore any functional significance. By comparing the koala prostate gland to eutherian sex accessory glands, we identified potential similarities and evolutionary divergences*.* Further analysis of secreted proteins across prostate segments provided insights into their distinct roles and allowed comparisons with eutherian prostatic secretions. Expanding our understanding of koala prostate segmentation not only deepens our knowledge of marsupial reproductive physiology but also has broader implications for assisted reproductive technologies in koala conservation. Furthermore, mapping the koala prostate proteome provides a foundation for future research into prostate-related pathologies in this threatened species.

## Methodology

### Chemicals

All chemicals used were purchased from Sigma-Aldrich (St. Louis, MO, USA) unless otherwise stated.

### Animals and tissue collection

All procedures were approved by the University of Queensland animal ethics committee (approval number 2021/AE001080). Mature male koalas (*n* = 6), *P. cinereus*) were sourced from RSPCA wildlife and Currumbin Wildlife Hospital from areas from Brisbane to Gold Coast, Queensland, Australia. Samples were collected from July to September, a period that corresponded to commencement of the koala breeding season and increasing secretion of androgen [18]. In all cases, animals were euthanized for welfare reasons by a lethal injection of pentobarbitone under gaseous (isoflurane) anesthesia. Individuals underwent clinical examination and exhibited no observable signs consistent with chlamydiosis and all samples were collected within 2 h postmortem. The prostate was excised and tissue was grossly sectioned from the most cranial and most caudal components of the gland, to sample the anterior and posterior segments, respectively. This segmentation is not visible macroscopically, and in order to ensure distinct sampling, the central prostatic segment, which comprises only 10% of the gland [12], was not sampled. The tissues were immediately snap frozen in liquid nitrogen and stored at −80°C until processing.

### Preparation of prostate tissue lysates

Tissues were lysed with 500 μL of lysis buffer (10 mM Tris, 2% (w/v) SDS, 1x Sigma Complete protease inhibitor) at room temperature for 15 min. Proteins were extracted using mechanical disruption with a tissue homogenizer (OMNI TH150) followed by sonication in a water bath (40 kHz, 30s). Following centrifugation (7500xg, 15 min), the supernatant was collected. Protein concentration was determined by a bicinchoninic acid (BCA) assay (Thermo Scientific, Pierce BCA Protein Assay Kit) according to manufacturer’s instructions and standardized across samples with MilliQ water.

Protein digestion was performed by filter-aided sample preparation (FASP). In total, 30 kDa Spin filters (Amicon, regenerated cellulose membrane) were prewashed (13 000*xg*, 15 min) with 200 μL of a urea and ammonium bicarbonate (UA) solution (8 M urea in 50 mM ammonium bicarbonate). Diluted lysate (200 μL containing 20 μg total protein) was added to the prepared spin filter (13 000*xg*, 15 min) then the sodium dodecyl sulfate (SDS) was removed by 2 washes with 200 μL of UA solution (13 000*xg*, 15 min each).

Proteins were reduced with 100 μL of dithiothreitol (DTT) solution (10 mM DTT in UA) for 30 min at room temperature and then centrifuged (13 000*xg*, 10 min). Proteins were then alkylated with 100 μL of iodoacetamide (IAA) solution (50 mM IAA in UA) for 30 min at room temperature in the dark and then centrifuged again (13 000*xg*, 10 min). Proteins were then subsequently washed with 100 μL of 8 M urea (13 000*xg*, 3 × 15 min) followed by 100 μL of 50 mM ammonia bicarbonate (13 000*xg*, 3 × 10 min). Proteins were digested overnight at 37°C with trypsin and 0.01% ProteaseMax (enzyme to protein ratio 1ug:50ug).

Peptides were eluted with 50 μL of 0.5 M NaCl (13 000*xg*, 3 × 15 min). A final clean-up was performed with using Millipore C18 ziptips (ZTC18S096) activated with 80% (v/v) acetonitrile (ACN), 0.1% (v/v) formic acid (FA) and then equilibrated with 0.1% (v/v) ACN, 0.1% FA. Peptides were washed three times (1% ACN, 0.1% FA) to remove contaminants, eluted with 10 μL of 80% ACN, 0.1% FA, dried under vacuum centrifugation and resuspended in 20 μL 0.1% formic acid for injection.

### Liquid chromatography–tandem mass spectrometry and SWATH acquisition

Isolated peptides were analysed by liquid chromatography–tandem mass spectrometry (LC–MS/MS), keeping individual biological replicates separate. Peptides were separated by reversed-phase LC on the Waters Acquity UPLC M-Class system. The peptides were separated using *a* Waters NanoEase HSS T3 column (100 Å, 1.8 μm, 300 μm × 150 mm) at a flow rate of 5 μL/min, with the column temperature set to 40°C. The LC conditions were set as follows: 0–0.5 min at 3%, 0.5–30.5 min at 3–30%, 30.5–34.5 min at 30–60%, 34.5–36.5 min at 60–97% then held at 97% for 4 min, followed by re-equilibration for 4 min. Pump A was 0.1% formic acid, 99.9% water (v/v), and Pump B was 0.1% formic acid in 99.9% acetonitrile (v/v).

Mass detection was performed with an ABSciex ZenoTof 7600 and operated with the OptiFlow Micro/MicroCal ion source. The parameters were set as curtain gas at 35 psi, CAD gas at 7 psi, ion source gas 1 was 20 psi and ion source gas 2 was set to 15 psi. The source temperature was 150°C with spray voltage set to 5000 V the DP Spread 80 V and collision energy 10 V. For Zeno-SWATH acquisitions, a Time of Flight Mass Spectrometry (MS TOF) scan was performed across 400–1500 m/z (0.1 sec). For MS2, variable windows spanning 399.5 m/z–750.5 m/z were chosen for fragmentation (0.013 sec). Fragment data was acquired across 140–1750 m/z using Zeno pulsing (threshold set to 100 000 cps) and the dynamic collision energy for MS/MS was used.

### Data processing and protein identification

Raw MS data was processed using DIA-NN software (v 1.9.2, University of Cambridge, UK). An in silico digest of the UniProt Koala (*P. cinereus*) proteome (downloaded November 2024) employing deep learning, was performed to create a spectral library. The search parameters used specified Trypsin as the enzyme used, allowing one missed cleavage. Carbamidomethylation of cysteine and N-term M excision were included as modifications. A false discovery rate (FDR) of 1% was applied at both the peptide and protein levels. Raw WIFF files were converted into MzML files using ProteoWizard (v 3.0, [19]). Spectra were mapped against the spectral library with mass accuracy set at 20 ppm.

The DIA-NN report file was converted to a workable file using the R package MSstats_labelfree_processing then processed through the R package MSstats [20] to perform filtering and statistical analysis of protein abundance in R studio (v 4.14.1). The dataProcess function was executed using default parameters, and the processed data underwent statistical analysis through pairwise comparisons with a linear mixed model. For the purpose of this study, a Log2FoldChange greater than 1.0 and less than −1.0 was used to determine a significant increase or decrease in expression levels and a *P*-value of less than 0.05 was used to demonstrate an increase in abundance of proteins.

### Bioinformatics analysis

DAVID (v 2023q4, https://davidbioinformatics.nih.gov/summary.jsp) was used to analyse gene ontology biological functions. A Bonferroni-adjusted *P*-value <0.05 was used to analyse enriched functions. R Studio (Version 2024.12.1 Build 563) was used with packages ggVennDiagram (v 1.5.2, [21]), tidyverse (v 2.0.0, [22]), RColorBrewer (v 1.1–3, [23]), FactoMineR (v2.11, [24]), factoextra [25], and missMDA [26] to create graphical interpretations of patterns in protein expression. Downstream pathways of the top 100 proteins in each segment (by label free quantification) were analysed using QIAGEN Ingenuity Pathway Analysis (IPA; QIAGEN Inc., https://digitalinsights.qiagen.com/IPA) using the core analysis function to generate canonical pathways based on expression fold change within the parameters of <−1 and > +1.

The proteome of the koala’s prostate was compared against the human and the mouse prostate proteomes. Protein Atlas (version 24.0, downloaded 1^st^ April 2025, https://www.proteinatlas.org/humanproteome/tissue/prostate) was the source of the human prostate proteome and supplied 14,288 gene IDs and ProteomeXchange Consortium dataset PXD003749 supplied the mouse prostate proteome (https://proteomecentral.proteomexchange.org, [27]. The mouse dataset was converted to official gene IDs using UniProt (https://www.uniprot.org/, [28]) and as with the koala dataset, duplicate gene IDs were removed and alternate ones were included, amounting to 2081 individual IDs. The koala prostate was also compared against the human prostate and the human seminal glands with Protein Atlas (version 24.0, downloaded 15 April 2025, https://www.proteinatlas.org/humanproteome/tissue/seminal+vesicle) being the source again for the seminal vesicle proteome, generating 13 204 gene IDs.

In order to identify secretory proteins from the koala prostate proteome, FASTA files were generated from the koala proteome list using UniProt [28]. Additional FASTA files were generated from the list of significantly increased proteins (*P*-value <0.05) with increased expression (anterior = log2 fold change >1, posterior = log2 fold change <1) in each segment. These datasets were then run through SignalP (v 6.0, Department of Health Technology, Denmark, https://services.healthtech.dtu.dk/services/SignalP-6.0/) to determine the presence of a signal peptide.

## Results

**Figure 2:**
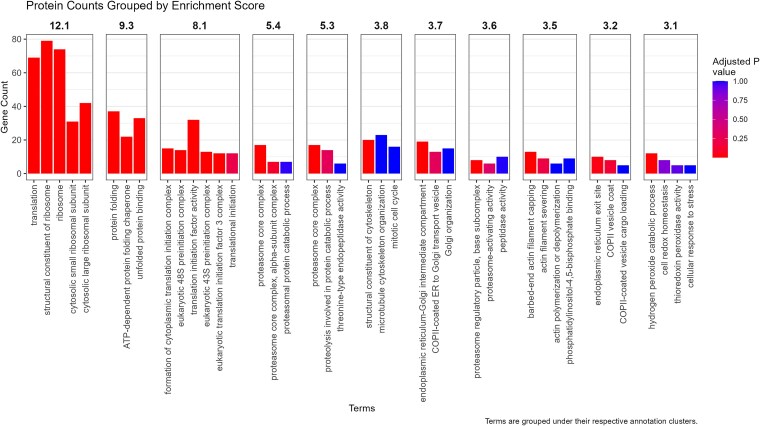
Protein counts grouped by enrichment score. DAVID functional analysis using gene ontology defines 11 annotation clusters which a significant enrichment score (>3). The adjusted *P*-value (Bonferroni) of each function is color graded.

Across the anterior and posterior prostate, DIA-ANN identified 17 510 peptides that mapped to 2708 known proteins at 1% FDR. A total of 2733 individual genes were identified among these proteins, accounting for any alternate gene variants corresponding to a single protein (Supplementary Data S1). Functional annotation of the entire prostate proteome in DAVID resulted in 53 clusters, 11 of which had high enrichment scores (>3) (Supplementary Data S2). The most enriched functional clusters in the koala’s prostate were related to protein translation, including ribosomes and protein folding, and proteasome function (Figure 2).

### Different segments of the koala proteome show modest proteomic differences, with functional impacts

**Figure 3:**
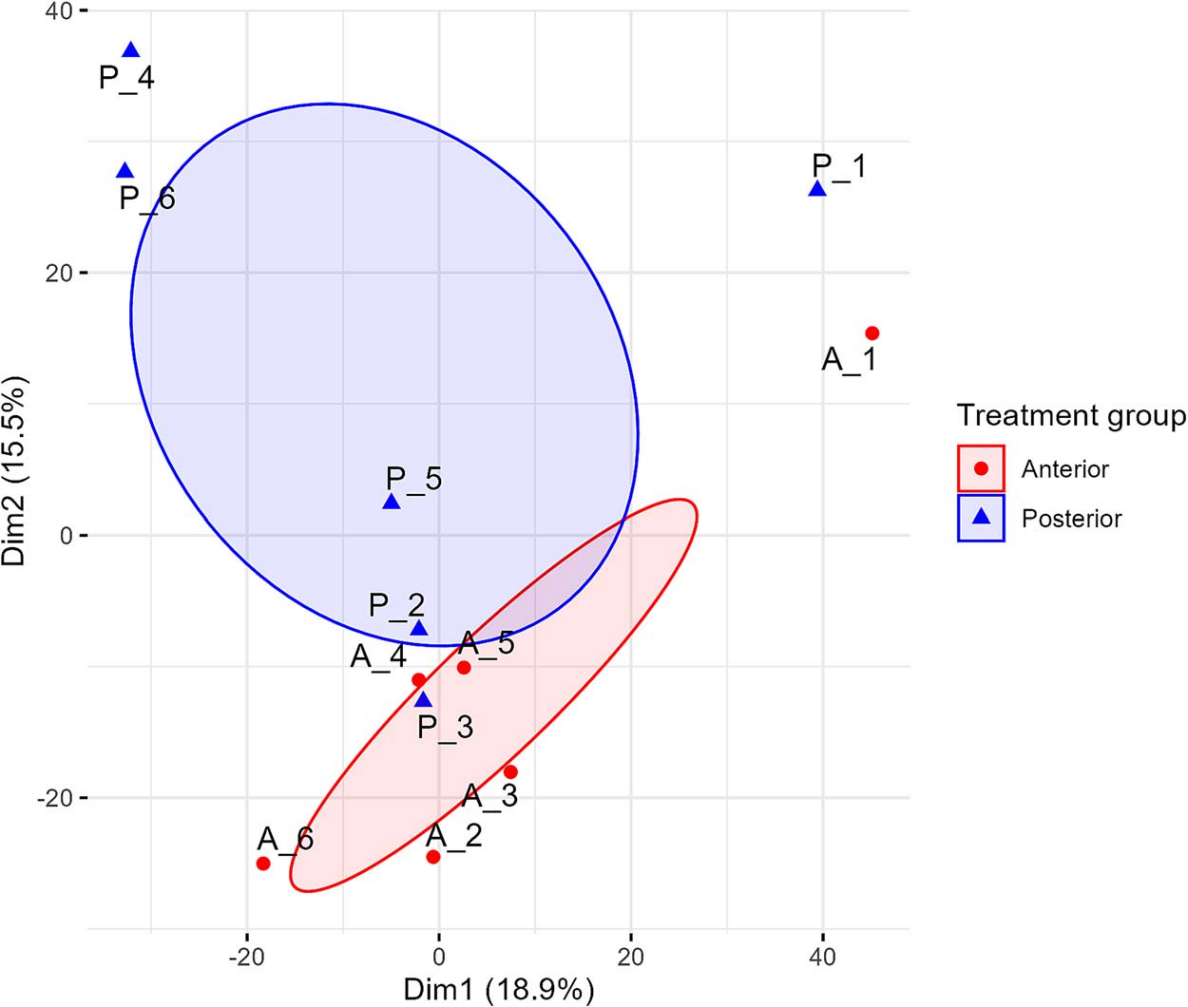
PCA of the koala prostate proteome illustrating distinct clustering between the anterior (red diamonds) and posterior (blue triangles) segments. Sample 1 (A_1, P_1) is positioned separately from the primary clusters, suggesting potential biological variation. Ellipses indicate within-group similarity and between-group distinction.

**Figure 4:**
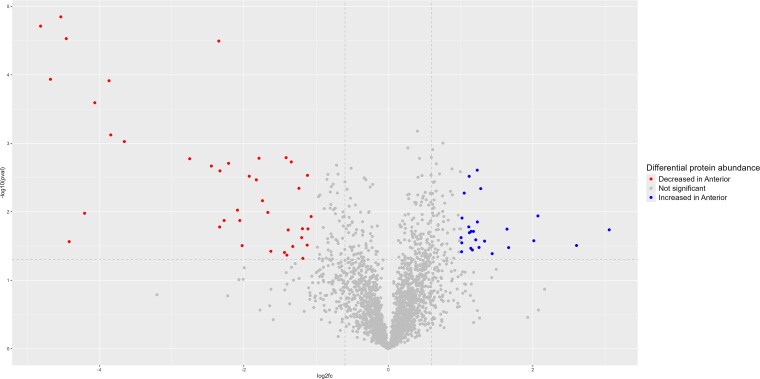
Volcano plot of *P*-value against log2 fold change in each prostate segment proteome. The increase and decrease in abundance of proteins in the AS with the inverse being true in the PS. Dotted gray lines represent the applied significance thresholds.

Sixty-five proteins (2.5%) were found to be significantly increased in abundance (log2 fold change >$\left|1\right|$, *P*-value <0.05), with 40 of these proteins upregulated in the anterior segment (AS) and 25 in the posterior segment (PS). A further 21 proteins were found in one tissue segment only; 6 in the AS and 15 in the PS (Supplementary Data S3). Proteins that were significantly different in the AS were compared against the PS. Principal component analysis (PCA) revealed distinct clustering between the anterior and posterior prostate segments, indicating discernible proteomic differentiation between these regions. Notably, Sample 1 is positioned separately from the primary clusters, suggesting potential biological variation within its segment (Figure 3). Figure 4 illustrates that although the most proteins are consistent across both segments, a small number showed significant differences. Specifically, there were more proteins significantly decreased in the AS, characterized by lower *P*-values and larger negative log2 fold change, and thus, conversely increased in the PS.

**Figure 5:**
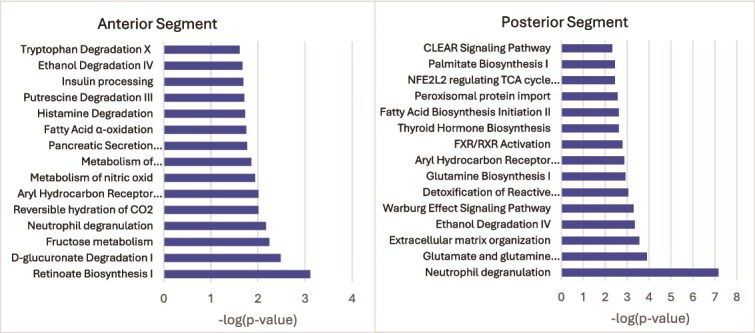
IPA of enriched proteins in each segment of the koala’s prostate. Top 15 canonical pathways identified in both the AS and PS using the list of enriched proteins from each segment.

Both the enriched proteins from each segment and those unique to one segment were analysed using IPA to identify any possible differences in pathway activation (Figure 5). Shared pathways included ethanol degradation, fatty acid processes, aryl hydrocarbon signaling and neutrophil degradation. In the AS, the top process was retinoate biosynthesis I and in the PS it was neutrophil degranulation.

**Figure 6:**
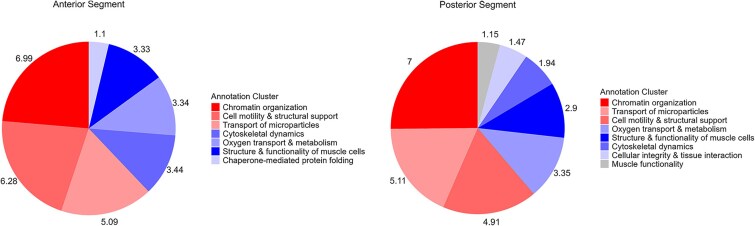
Functional annotation clusters by segment of the koala prostate gland. The top 100 proteins identified in each segment analysed by DAVID Gene Ontology demonstrated 7 enriched functions in the AS and 8 in the PS.

The top 100 most abundant proteins of each segment, measured by median label free quantification values, were analysed by DAVID to determine any functional differences between the anterior and PSs of the prostate. Many of the enriched functions were similar with slight variations in the intensity of enrichment (Figure 6). Protein folding was a functional cluster in the anterior but did not appear in the PS. Cellular integrity was clustered in the posterior but not in the anterior. In the AS, the enriched functions included chromatin organization, cell mobility, transport of microparticles, cytoskeletal dynamics and cell cycle progression, oxygen transport and the structure and function of muscle cells. In the PS, the enriched functions were chromatin organization, transport of microparticles, cell motility and muscle function.

### Cross species comparison reveals significant conservation of sex accessory gland proteomes

**Figure 7:**
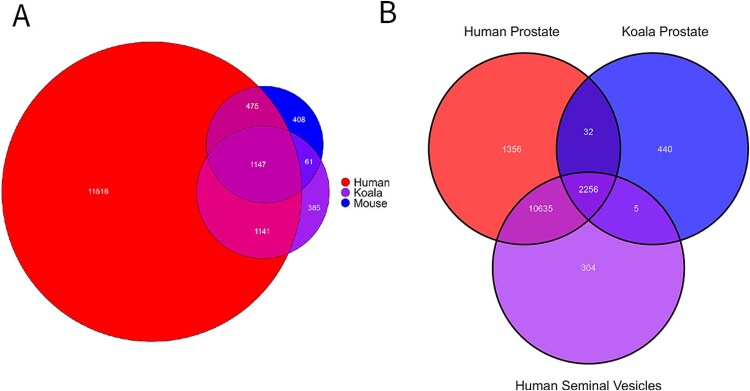
Venn diagrams comparing proteome of sex accessory glands with conserved proteins represented as a count in the overlapping regions. (A) Comparison of the human, mouse and koala prostate proteome. Data from this study was used to compare conserved and unique proteins using gene IDs as a common annotation. This koala prostate proteome was compared against the human prostate proteome (version 24.0, downloaded 1 April 2025, https://www.proteinatlas.org/humanproteome/tissue/prostate) and the mouse prostate proteome (https://proteomecentral.proteomexchange.org, [42]). (B) Comparison of the proteome of the koala prostate, human prostate and human seminal vesicles. Data generated in this study were compared against published proteomes for the human prostate (version 24.0, downloaded 1 April 2025, https://www.proteinatlas.org/humanproteome/tissue/prostate) and human seminal vesicles (version 24.0, downloaded 15 April 2025, https://www. https://www.proteinatlas.org/humanproteome/tissue/seminal+vesicle) to identify the total number of conserved proteins across species based on Gene ID.

Comparative genomic analysis of the koala (*P. cinereus*) prostate gland revealed a close similarity to the human (*Homo sapiens*). Of the 2733 identified koala prostate gene IDs, 2288 (83.71%) showed homology with the human prostate, whereas only 1208 gene IDs (44.2%) were shared with the mouse (*Mus musculus*) (Figure 7A). Although the greater alignment between the human and koala prostate proteomes may largely reflect the extensive proteomic data available for humans, the observed overlap nonetheless highlights notable molecular similarities between the two species. Expectantly, there was a significant overlap between the prostate and the seminal vesicles of the human, which in turn, resulted in a substantial overlap with the koala prostate and human seminal vesicles (Figure 7B). However, there were 5 genes common to the koala prostate and human seminal vesicles that do not appear in the human prostate: AKR1E2, BBOX1, GDA, LRRC43, and PON3. The functions of these genes and their encoded proteins have been listed in Supplementary Data S3.

### Analysis of secreted proteins

Three hundred and fifty-four (13%) of the total 2708 identified proteins in the koala prostate were predicted to be secreted. From the list of proteins that were significantly different in each segment (Supplementary Data S4), there were 7 secretory proteins in the AS and 14 in the PS, the functions of which have been listed below in Table 1. In analysing enriched proteins only, there was no commonality with either the human seminal vesicles or human prostate gland secreted proteins.

## Discussion

Utilizing LC–MS/MS, this study successfully generated the first proteomic profile of the koala prostate gland, offering valuable insights into its functional composition. Furthermore, it is the first report on the prostate gland proteome in the mammalian infraclass *Marsupalia*, a taxon known to have a prominent prostate gland both in size and in functionality [29]. While the proteomic analysis of the koala prostate gland excluded the central segment (CS), this segment comprises only 10% of the prostate gland and it is thought to have lesser role in the production of secretions [12]. Proteomic profiling of the koala prostate revealed that the whole gland was highly enriched in functions of translation, ribosomes and protein folding, relative to all proteins detected. This reflects a strong demand for protein synthesis, modification and folding, which is synonymous with high secretory activity [30] and aligns with the prostate’s role in the production of seminal plasma. Furthermore, by profiling the proteome of prostates free from clinical signs of pathology, we have created a baseline for future research to identify changes associated with reproductive disease in koalas. Further research investigating the proteomes of *Chlamydia* spp infected koala prostates may now be able to isolate deviations in expression patterns and identify biomarkers linked to chlamydiosis related pathology. Establishing this reference framework is essential for advancing pathology research and for improving veterinary interventions for reproductive disease in koalas.

In comparing the proteins present in each segment, the PCA (Figure 3) demonstrated distinct clustering, suggesting biologically distinct compositions in the anterior and posterior prostate. The separation of sample 1 from the primary clusters may be attributed to an increased post-mortem interval, as the animal perished enroute to the hospital. However, studies suggest that protein composition remains largely stable within 24 h post-mortem [31], so it is not clear if this is the determining factor. While wild koalas exhibit a documented breeding season from spring to early autumn, physiological changes such as increased dispersal, elevated testosterone levels, and body mass typically begin in late winter [18]. All samples in this study were collected during this period, largely due to the heightened availability of individuals, as breeding season coincides with peak wildlife trauma admissions. Although no seasonal anatomical changes have been documented in the koala prostate gland [18], it would be valuable to compare glands collected outside the breeding season to assess whether there is any functional differences.

Overall, there was less differentiation in protein composition than the gross morphology or histochemical appearance of the prostate would imply, with only 86 significantly different proteins. Thirty-one proteins were significantly more increased in the AS (1% of all proteins identified) and 55 proteins (2%) in the PS. Functional analyses depicted highly enriched functions related to muscle structure and operation in both segments. This is in line with expectations given the physiological need for the prostate to facilitate fluid expulsion. Additionally, microparticle transport is a prominently enriched function in both segments. This is noteworthy given that prostatic bodies, hypothesized to be akin to human prostasomes (extracellular vesicles), have only been observed in the AS [12]. The canonical pathways as identified by analysing the significantly increased proteins in each segment were quite different (Figure 5). Pathways in the PS were more broadly focused on energy production for cells and dealing with the associated byproducts. The AS appeared to have a focus on degradation of waste products and regulating the cell environment. Retinoate biosynthesis (Figure 5 Anterior) is well documented as having a significant role in reproduction, including male germ cell development, fertilization and embryonic implantation [32]. The possibility of a fructose metabolism pathway in the AS is intriguing, especially considering that marsupial spermatozoa typically utilize glycogen or N-acetylglucosamine as energy substrates [33]. Brooks et al. [34] identified fructose in the prostate gland of the southern hairy-nosed wombat (*Lasiorhinus latifrons*), and given the phylogenetic proximity of this species, it is plausible that the koala’s prostate may also produce fructose. The relative subtlety of the proteomic difference in the two segments compared to the pronounced morphological distinction is a noteworthy observation in itself. Future investigations could benefit from excluding documented accessory gland housekeeping proteins during bioinformatic analyses, as their presence may obscure biologically meaningful variation. However, at present, the number of reliably identified housekeeping genes specific to accessory gland or secretory tissues remains limited.

Looking at the koala prostate in an interspecies comparison, the results are consistent with previous research suggesting similarities between the koala and human prostate glands [15, 35]. Our work further demonstrates the koala’s prostate has similarities with the human prostate, with 84% of identified proteins in common. In contrast, only 56% of identified proteins matched those present in the mouse prostate. This is partially explained by the size of the mouse prostate proteome being significantly smaller than that which has been mapped in the human prostate. Both the mouse and the human possess prostates that are also segmented to some degree, although in the mouse, these segments are classified as lobes and are geographically distinct [36]. In humans, zonal architecture is more akin to the koala’s segmentation, being not discernible externally, but histologically differentiated [37]. Immunohistochemistry analysis of basal and luminal cell ratios has highlighted possible similarities between the CS of the koala prostate and the transitional zone of the human prostatic gland, as well as the AS and the human peripheral zone [12]. Skerrett-Byrne [15] also demonstrated a significant similarity between the proteome of human and koala prostate extracellular vesicles, with an 82.5% overlap. These findings further support the proposition that the koala (*P. cinereus*) may serve as a valuable research model for human reproductive biology, not in terms of a classical laboratory species but as an example of a naturally infected host for chlamydial prostatitis [5]. By leveraging naturally infected individuals, the koala may be used for epidemiological studies that reflect real-world transmission dynamics and to analyse host-pathogen interactions, and tissue-specific responses to *Chlamydial* infection within the prostate gland [35].

Alongside the alignment between the human and koala prostate gland, there was also strong similarity between human seminal vesicles and the koala prostate. Interestingly, there were 5 proteins unique to the koala prostate and human seminal vesicles, that were not present in the human prostate proteome (AKR1E2, BBOX1, GDA, LRRC43, PON3). As previously stated, koalas, like all marsupials, lack seminal vesicles and therefore it is useful to analyse the functions of these proteins. AKR1E2 plays a key role in catalyzing energy substrates, specifically facilitating the conversion of 1,5-anhydro-D-fructose (AF) into 1,5-anhydro-D-glucitol, a glycogen metabolite [38]. BBOX1 encodes for the enzyme that catalyzes the formation of L-carnitine, a key substance in fatty acid metabolism [39]. In humans, L-carnitine is present in high volumes in seminal plasma, although it is epididymal derived, and L-carnitine-mediated oxidation of fatty acids has a strong correlation with spermatozoa motility [40]. Furthermore, BBOX1 was significantly increased in the koala’s posterior prostatic segment, further suggesting that this segment may have a role in energy metabolism for spermatozoa.

While in human male, seminal vesicles are responsible for the production of the energy substrate for spermatozoa [41], it is assumed that the prostate performs the same role in marsupials [33]. Our study adds further evidence to the theory that this the case in the koala, however, it is unclear what that this substrate might be. While polysaccharides (such as glycogen) have been detected in the koala’s prostate [12], whether koala spermatozoa rely exclusively on glycogen as an energy substrate remains uncertain.

Finally, a notable proportion (13%) of the proteins found in the koala prostate appear to be secretory. While the number of predicted secretory proteins enriched in the PS (14) was higher compared to the AS (7), this study was conducted on whole tissue rather than solely secretory fluid, the relative volume of secretions in each segment remains undetermined. Future studies investigating segmentation in the koala prostate would benefit from complementary proteomic analyses of both the tissue and the segment-specific secretions, allowing for a clearer distinction between secreted and epithelium protein profiles. Nevertheless, analysing the functions of these enriched secretory proteins is useful given previous research has demonstrated significant morphological differences in each segment’s secretory material [12]. In analysing the individual functions of the 7 secreted proteins in the AS and the 14 in the PS (Table 1), it appears that the anterior prostate’s secretions are directed toward maintaining the Golgi apparatus, regulating protein degradation, extracellular communication, calcium binding, sorting prohormones and the production of mucus. The secretions in the PS, however, are focused on energy metabolism, immune response, protein breakdown, neuroendocrine peptide production and cell communication and adhesion.

Furthermore, in considering enriched proteins only in the human prostate, and seminal vesicles, the lack of conserved secretory proteins across all three organs may highlight that the composition of prostatic fluid could be quite different between species. A more refined analysis of prostatic fluid rather than whole tissue may present a different outcome as this study was restricted to comparing enriched secretory proteins only. Nevertheless, these findings underscore the functional specialization of the secretions produced by each segment, and how they may contribute to the broader physiological roles of the koala prostate.

## Conclusion

This is the first study that examines the secretory and functional differences in prostatic segmentation, a phenomenon that appears to be common to every marsupial species studied to date. Our data reinforces the theory that the prostate gland of the koala is functionally adapted to fulfil the role of alternate accessory glands such as the seminal vesicles which marsupials do not possess. We also noted that the koala prostate has a remarkable similarity to that of the human prostate gland. Given zonal segmentation is also present in humans, publication of an in-depth proteomic analysis of each zone, including secretory functions, would be beneficial to examine any further similarities.

Further understanding of the functional role of the koala’s sex accessory glands would benefit from a similar proteomic analysis of the koala bulbourethral glands. It would be useful to compare the functions of these sex accessory glands against the prostate to confirm the theory that the koala’s prostate gland is the primary producer of seminal plasma and has a more active role in successful reproduction. Proteomic analyses of sex accessory glands are fundamental to the study of reproduction. In a species threatened by reproductive pathology, a comprehensive proteomic profile aids in studying disease while informing conservation through assisted reproductive technologies.

## Supplementary Material

Supplementary_data_1_ioaf209

Supplementary_data_2_ioaf209

Supplementary_data_3_ioaf209

Supplementary_data_4_ioaf209

## Data Availability

The data underlying this article is available in the article and in its online supplementary material. The data that support the findings of this study will be openly available in ProteomeXchange Consortium (http://proteomecentral.proteomexchange.org) via the PRIDE partner repository with the dataset identifier PXD067256.

## Declaration of originality

This research is the original work of all authors.
